# Comparative Abilities of Fasting Plasma Glucose and Haemoglobin A1c in Predicting Metabolic Syndrome among Apparently Healthy Normoglycemic Ghanaian Adults

**DOI:** 10.1155/2019/2578171

**Published:** 2019-07-24

**Authors:** Nafiu Amidu, William Kwame Boakye Ansah Owiredu, Lawrence Quaye, Peter Paul Mwinsanga Dapare, Yussif Adams

**Affiliations:** ^1^Department of Biomedical Laboratory Science, University for Development Studies, Tamale, Ghana; ^2^Department of Molecular Medicine, Kwame Nkrumah University of Science and Technology, Kumasi, Ghana

## Abstract

There are arguments as to whether haemoglobin A1c (HbA1c) better predicts Metabolic syndrome (MetS) than fasting plasma glucose. The aim of the study was to explore the comparative abilities of HbA1c and Fasting plasma glucose (FPG) in predicting cardiometabolic risk among apparently healthy adults in the Tamale metropolis. This study was a cross-sectional study conducted in the Tamale metropolis from September, 2017, to January, 2018, among one hundred and sixty (160) apparently healthy normoglycemic adults. A self-designed questionnaire was administered to gather sociodemographic data. Anthropometric and haemodynamic data were also taken and blood samples collected for haemoglobin A1c (HbA1c), fasting plasma glucose (FPG), and lipid profile. MetS was classified using the harmonised criteria as indicated in the joint interim statement (JIS). Out of the 160 participants, 42.5% were males and 57.5% were females. FPG associated better with MetS and other cardiovascular risk markers, compared to HbA1c. FPG had the largest area under curve for predicting MetS and its components. This study shows a stronger association between FPG and MetS compared with haemoglobin A1c; it also provides evidence of a superior ability of FPG over HbA1c in predicting MetS and other adverse cardiovascular outcomes in apparently heathy normoglycemic individuals.

## 1. Background

Metabolic syndrome (MetS) is a set of closely associated cardiometabolic risks [1], like obesity, dyslipidemia, hypertension, and hyperglycemia and is seen as a powerful indicator of diabetes and cardiovascular disease (CVD) [2, 3]. The prevalence of metabolic syndrome continues to be on the rise; this is in part as a result of rapid urbanization with the related variations in nutrition and physical activity [4]. Worldwide the prevalence of metabolic syndrome has been reported as being between 10% and 84% [5]. In Africa, prevalence of 2.1% to 34.7% has been reported in several studies from around the continent [6, 7]. In Ghana, a prevalence of metabolic syndrome between 6% and 21.2% has been reported [8] using different criteria.

Haemoglobin A1c (HbA1c), a result of nonenzymatic glycosylation of the *β*-chain of haemoglobin, is made in proportion to the rise in blood glucose levels. It has been considered a preferable tool since HbA1c assay has superior technical advantages compared to the estimation of plasma glucose; it can be measured in the nonfasted state and has greater reproducibility than fasting glucose [9, 10]. HbA1c is a set-up marker of long haul glycemic control in individuals with diabetes mellitus (DM), and increased HbA1c levels are linked with an increased risk for later microvascular and macrovascular illness [11].

The fasting plasma glucose (FPG) cut-off figure for MetS may differ among various populaces. There are numerous reports recommending that HbA1c is superior to FPG in forecasting cardiometabolic risk even in nondiabetic individuals [12–14], with many others proposing that HbA1c may be an essential marker for MetS, but it stays a controversy [15–17]. However, HbA1c may be influenced by various haematologic, genetic, and disease-related factors [18]. The most important factors globally affecting HbA1c levels are some anaemias, haemoglobinopathies, and disorders linked with increased red blood cell turnover like malaria [9, 19].

A 1% rise in HbA1c raises the risk of CVD by 18% and positive relation between CVD and HbA1c has been shown in nondiabetic individuals even within normal values of HbA1c [20]. Many population-based studies from Western nations have investigated the link between HbA1c and the risk of CVDs (MetS) among nondiabetics [14, 21, 22], while only a few studies were from Africa and for that matter Ghana has examined this issue. Moreover, there is scarce evidence about whether or not adding HbA1c to other possible risk factors improves the ability to predict the Metabolic syndrome.

Previous studies have related HbA1c to glucose and weighed the option of replacing glucose with HbA1c for the criterion or adding HbA1c as an extra criterion for diabetes [17, 23–26]. However, data on the use of HbA1c as an indicator of MetS particularly in nondiabetic people are scanty and inconclusive, with some studies supporting the possible use of HbA1c as a marker for MetS, while other studies show divergence [15, 24, 27, 28]. While some studies have observed the importance of haemoglobin A1c in MetS, few have studied it in individuals with normal glucose levels. The aim of the study was to explore the comparative abilities of HbA1c and FPG in predicting metabolic syndrome in apparently healthy normoglycemic adults within the Tamale metropolis of Ghana.

## 2. Methods

### 2.1. Subjects

This study was a cross-sectional study conducted among apparently healthy adults (18 years and above) with no history of diabetes within the Tamale metropolis from September, 2017, to January, 2018.

#### 2.1.1. Exclusion Criteria

Diabetics, hypertensives, persons treating diabetes or hypertension, persons with a fasting blood glucose >7.0 mmol/l or HbA1c ≥6.5% at the time of the study, pregnant women, persons showing signs of any acute illnesses, and persons with other chronic diseases were excluded from this study.

#### 2.1.2. Sample Size

The minimum sample size for the study was calculated to be 105 adults, based on the assumption that 7.4% of the normal adult populations have metabolic syndrome [29], with an expected difference of 5% between the sample and the general population and a type I error (*α*) of 0.05.

This study was limited to only apparently healthy adults who answered at least 75% of the questions in the questionnaire and did not have an FPG of >7.0 mmol/l or an HbA1c of >6.5; hence, the sample size was recalculated to adjust for any possible loss of respondents. Assuming a response rate of 90%, the sample size was recalculated to be approximately 117. One hundred and twenty (120) participants were therefore targeted for this study.

### 2.2. Data Collection

#### 2.2.1. Sociodemographic and Anthropometric Data

A self-designed semistructured questionnaire was administered to consented study participants for sociodemographic data. Weight to the nearest 0.1 kg was measured using a digital flat floor weighing scale (with weighing capacity of 250 kg) manufactured by SECA (Hamburg, Germany) and height to the nearest 1 cm was measured using a portable microtoise (measuring range: 0 cm to 220 cm) manufactured by SECA. Waist circumference (to the nearest centimetre) was measured with a Gulick II spring-loaded measuring tape (Gay Mill, WI) midway between the inferior angle of the ribs and the suprailiac crest. Hip circumference was measured as the maximal circumference over the buttocks in centimetre.

#### 2.2.2. Blood Pressure

Blood pressure was measured in sitting position, with a sphygmomanometer cuff and a stethoscope. Measurements were taken from the left brachial artery after subjects had been sitting for at least five (5) minutes in accordance with the recommendation of the American Heart Association [30]. Triplicate measurements were taken with a five (5) minute rest interval between measurements and the mean value was recorded to the nearest 2.0 mmHg.

#### 2.2.3. Sample Collection, Preparation, and Analysis

Ten milliliters (10 ml) of venous blood sample was collected under strict aseptic conditions from each participant in the morning between 07.00 and 09.00 GMT into fluoride oxalate tube, Serum Separator Tubes (SST), and ethylenediaminetetraacetic acid (EDTA) anticoagulated tube (Becton Dickinson, Rutherford, NJ), after an overnight (8-12 hours) fast. Samples in the fluoride oxalate tubes were centrifuged and plasma was used for glucose measurement (within 2 hours after sample collection) using the Glucose oxidase peroxidase (GOD-POD) method whilst samples in the SST were centrifuged at 3000 g for 5 minutes and the serum was aliquoted and stored in cryovials at a temperature of -80°C until time for biochemical assays. Lipid profile and fasting blood glucose levels were determined using the Mindray BS-240 Chemistry Analyser (Mindray, China); MedSource Diagnostics reagents were used in all of these assays. The anticoagulated (EDTA) blood was used for the HbA1c Assay using the MedSource Diagnostics reagents for Glycosylated Haemoglobin (A1-fast fraction) test kit which uses the Cation Exchange Method. For the within run (intra-assay) precision, a % CV was 2.7 in normal HbA1c samples and 1.7 in elevated HbA1c samples was quoted while for the run to run (Inter run) precision a % CV was 4.1 for normal samples and 4.6 for elevated samples were quoted by manufacturers. Samples from subjects with haemoglobinopathies or decreased erythrocytes survival times may show incorrect results. This method is not listed in the 2019 National Glycohemoglobin Standardization Program (NSGP) method traceability list.

### 2.3. Definitions of Metabolic Syndrome

#### 2.3.1. Metabolic Syndrome: Harmonised Criteria by the Joint Interim Statement (JIS)

Metabolic syndrome was defined to include individuals with any three or more of the following five components: (1) abdominal obesity (waist circumference, Male ≥94, Female ≥80), (2) high triglyceride ≥ 1.7 mmol/L (150 mg/dl), (3) low HDL-C: Male< 1.0, Female <1.3 mmol/L, (4) High BP (systolic BP ≥ 130 mm Hg or diastolic BP ≥ 85 mm Hg or treatment of hypertension), and (5) high fasting glucose ≥ 5.6 mmol/l [31].

### 2.4. Statistical Analysis

All analyses were performed using MedCalc® version 10.2.0.0 (www.medcalc.be) for windows and GraphPad version 6.0, San Diego, California, USA. Unpaired T-test was used to compare continuous variables. Association between variables was assessed with linear regression analysis. Receiver Operator Characteristics (ROC) was used to compare the relative abilities of various parameters to predict MetS and other cardiovascular risk factors. In all statistical analyses, a p value of <0.05 was considered significant.

## 3. Results

### 3.1. General Characteristics of Studied Population

A total of 160 complete questionnaires were analysed, of which 68 (42.5%) were males and 92 (57.5%) females. Subjects with metabolic syndrome were significantly older than subjects without the metabolic syndrome. The average HbA1c and FPG of the study population were 4.8±1.2% and 4.95±0.92 mmol/L, respectively. These parameters were higher in respondents with MetS; however, only the difference in FPG was statistically (p<0.001) significant as shown in Table 1

### 3.2. Biochemical Parameters of Studied Population Stratified by Gender

Table 2 summarises the biochemical parameters of the studied population stratified by gender. Female respondents were older (43.8±14.3 years) than the male (41.4±14.8 years) but this was not statistically significant. Female respondents with MetS however were significantly older than those without MetS. In females only, FPG was significantly higher in MetS as shown in Table 2.

### 3.3. Biochemical Characteristics according to MetS Score

Table 3 shows the anthropometric and biochemical variations in MetS scores. Generally, FPG significantly showed an increasing trend while moving from a score of 0 to a score of 3 or more.

### 3.4. Association between HbA1c, FPG, Lipid Parameters, and MetS Score

A linear regression between HbA1c, FPG, and selected cardiometabolic risk is shown in Table 4. HbA1c had significant positive association with triglyceride and VLDL-c. A percentage increase in HbA1c results in a 0.12 mmol (r^2^=0.03, p<0.05) increase in Triglyceride and 0.05 mmol (r^2^=0.03, p<0.05) increase in VLDL-c. FPG however showed significant positive association with SBP, DBP, total cholesterol, triglyceride, and VLDL-c. A 1 mmol/L increase in FPG is associated with an increase in 0.33 mmol/L (r^2^=0.05, p<0.01) of total cholesterol, 0.21 mmol/L (r^2^=0.05, p<0.01) of triglyceride, and a 0.10 mmol/L (r^2^=0.05, p<0.01) increase in VLDL-c.

### 3.5. Receiver Operator Characteristics (ROC) for HbA1c and FPG in the Studied Population

**Figure 1:**
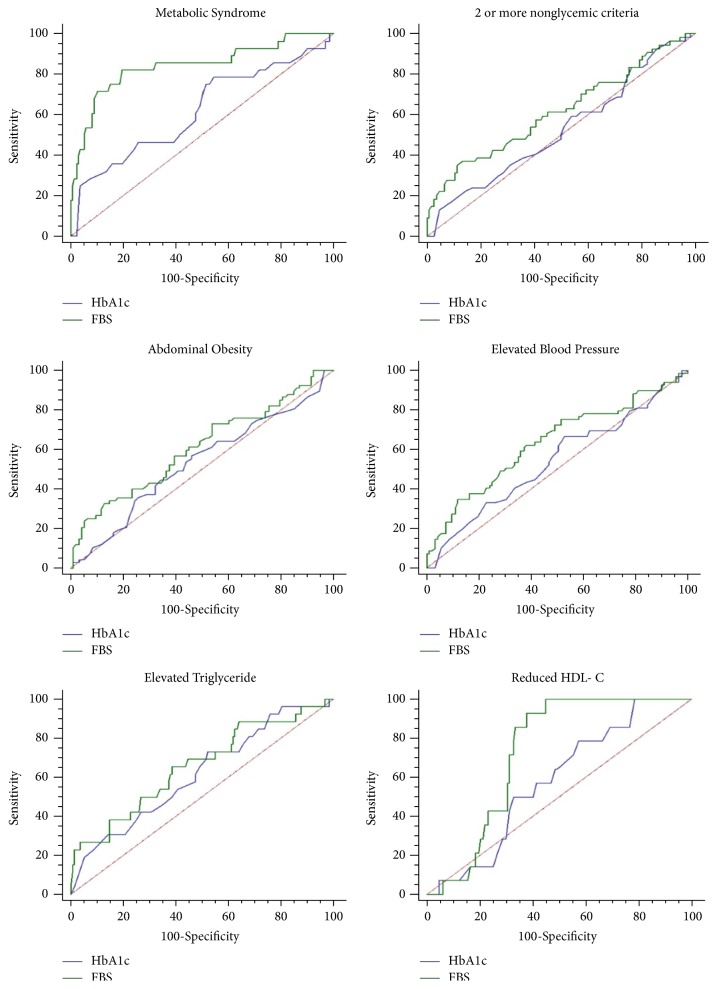
ROC curves for MetS. Compared are the relative abilities of HbA1c and FPG to identify respondents with MetS and its components.

The ROC curves and the Area under Curve (AUC) between HbA1c and FPG against MetS and its individual components are shown in Figure 1 and Table 5. FPG had the largest AUC for all variables assessed, that is, MetS, 2 or more nonglycemic components, abdominal obesity, elevated BP, elevated triglyceride, and reduced HDL-c (Table 5).

## 4. Discussion

The role of impaired glucose metabolism in the pathogenesis of MetS and its adverse effects on CVDs and diabetes outcomes has been well documented [32, 33]. Hyperglycemia is known to compound the problem in MetS through the formation of advanced glycation end products [34].

Fasting plasma glucose and haemoglobin A1c measurements have been used over the years in the diagnosis of impaired glucose metabolism. However, proper consensus has not been reached about which there is a better diagnostic tool, associates better with cardiometabolic risk, and can be used as a predictive tool for MetS, especially among normoglycemic individuals. Some studies have shown that haemoglobin A1c associates better with cardiometabolic risk [16, 24, 35].

This study however found that haemoglobin A1c does not associate better with cardiometabolic risk and has no superior ability in predicting the presence of MetS among a normoglycemic northern Ghanaian population. Succurro and Marini [23] pointed out that the classification of MetS using a HbA1c criterion instead of glucose performed worse in detecting some subjects who still had an unfavourable cardiometabolic risk profile. Several other studies have reported similar findings, especially among a normoglycemic population [36].

The adverse effects of impaired glucose metabolism and diabetes are as a result of the elevated glucose levels and not elevated levels of haemoglobin A1c which is only reflective of a chronic exposure to high plasma glucose concentration [37]. There is evidence that each of the glycemic measures used to identify prediabetes represents a different domain of glucose metabolism. While FPG reflects basal dysglycemia, HbA1c reflects chronic exposure to basal and postprandial hyperglycemia [37]. A nonlinear relationship between glycemia and the haemoglobin A1c in normoglycemic populations has been observed in a number of studies which have shown that glycemia may be a less important determinant of hemoglobin glycation and that other factors operate to produce consistent changes in HbA1c. Potential explanations for this variation in hemoglobin glycation at or near normal glucose levels have focused on interindividual variation in red cell turnover [38], differences between the intraerythrocyte and extraerythrocyte environment [39], and genetic variation in hemoglobin glycation [40]. This means that, in a normoglycemic population, estimation of glucose levels will correlate better with adverse cardiometabolic outcomes than haemoglobin A1c as shown in the present study.

In this study, though there was no estimation of haemoglobin glycation index (HGI) and data on HGI among African populations that remain sparse, some studies in developed countries have revealed a lower glycation index among African Americans and Caucasians compared with Hispanics [41]. This means that, even at elevated glucose levels, formation of haemoglobin A1c among the population in the present study may have been slow and hence haemoglobin A1c did not reflect the glycemia. Hence, the subsequent absence of association between glycation and the cardiometabolic risk factors and its inability to properly predict MetS and its components compared to Fasting Blood Glucose.

Various combinations of haemoglobin variants C and S have been reported to falsely lower the values of HbA1c. The reported higher frequencies of these variants especially haemoglobin C among sub-Saharan Africans [42, 43] could be linked to the nonperformance of HbA1c in this study, and therefore the impact of haemoglobinopathies in this current study cannot be underestimated especially among a study population of predominantly Northern descent where the prevalence of the haemoglobin C has been shown to be appreciable [44].

## 5. Conclusion

This study demonstrates that, in a normoglycemic population, FPG associates better with Metabolic syndrome and other cardiometabolic risks than HbA1c and that fasting blood glucose estimation is shown to be the best predictor of MetS and its components among an apparently normoglycemic population.

### 5.1. Limitations

The estimation of haemoglobin A1c in this study was limited to only one method (Medsource Ozone Biomedicals Pvt., Ltd.) which is not listed on the 2019 NSGP certified methods list.

## Figures and Tables

**Table 1:** Biochemical parameters of studied population stratified by MetS.

Variables	Total	No MetS	MetS	P value
	(n=160)	(n=132)	(n=28)	
Age (years)	42.8±14.5	41.6±14.6	48.2±12.9	0.030
HbA1c (%)	4.8±1.2	4.8±1.2	5.2±1.3	0.080
FPG (mmol/L)	5.0±0.9	4.8±0.9	5.8±0.7	<0.001

HbA1c: Haemoglobin A1c and FPG: Fasting Blood Glucose. Data are presented as mean ± SD and compared using T-test.

**Table 2:** Biochemical parameters of studied population stratified by gender.

Variables	Male			Female		
	Total	No MetS	MetS	Total	No MetS	MetS
	(n=68)	(n=60)	(n=8)	(n=92)	(n=72)	(n=20)
Age (years)	41.4±14.8	41.8±15.2	38.6±10.9	43.8±14.3	41.5±14.2‡‡	52.0±11.8
HbA1c (%)	4.8±1.3	4.8±1.3	5.1±1.1	4.9±1.2	4.7±1.1	5.3±1.3
FPG (mmol/L)	5.0±0.9	5.0±0.9	5.6±0.7	4.9±1.0	4.6±0.9‡‡‡	5.8±0.7

HbA1c: Haemoglobin A1c and FPG: Fasting Blood Glucose. Data are presented as mean ± SD and compared using T-test. ‡Comparing females with MetS with females without MetS. ‡Comparison is significant at the 0.05 level, ‡‡Comparison is significant at the 0.01 level, and ‡‡‡Comparison is significant at the 0.001 level.

**Table 3:** Biochemical characteristics stratified by MetS component score.

Variable	MetS score					
	0 (n=42)	1 (n=52)	2(n=38)	≥3(n=28)	F Value	P Value
Age (years)	34.6±11.8	42.4±14.2	48.3±15.0	48.2±12.9	8.66	<0.001
HbA1c (%)	4.8±1.1	4.8±1.3	4.6±1.2	5.2±1.3	1.24	0.297
FPG (mmol/L)	4.43±0.78	4.9±0.9	5.0±0.9	5.8±0.7	14.46	<0.001

HbA1c: Haemoglobin A1c and FPG: Fasting Blood Glucose. Data are presented as mean ± SD and compared using One-way ANOVA.

**Table 4:** Linear regression analysis between HbA1c, FPG, and selected indicators of cardiometabolic risk factors.

Variable	HbA1c		FPG	
	*β*	r^2^	*β*	r^2^
SBP (mmHg)	0.58	0.00	4.10*∗∗*	0.06
DBP (mmHg)	-0.26	0.00	2.17*∗*	0.03
HbA1dc-Dcct (%)	-	-	0.12	0.01
FPG (mmol/L)	0.07	0.01	-	-
Total cholesterol (mmol/L)	0.07	0.00	0.33*∗∗*	0.05
Triglyceride (mmol/L)	0.12*∗*	0.03	0.21*∗∗*	0.05
HDL-c (mmol/L)	0.00	0.00	0.22	0.04
LDL-c (mmol/L)	0.02	0.00	0.01	0.00
VLDL-c (mmol/L)	0.05*∗*	0.03	0.10*∗∗*	0.05
MetS score	0.06	0.00	0.56*∗∗∗*	0.20

*∗*Regression is significant at the 0.05 level, *∗∗*regression is significant at the 0.01 level, and *∗∗∗*regression is significant at the 0.001 level.

**Table 5:** AUC for HbA1c and FPG in predicting MetS and its components.

Variable	HbA1c	FPG
MetS	0.62(0.54-0.69)	0.84(0.78- 0.89)
2 or more nonglycemic criteria	0.53(0.45- 0.61)	0.62(0.54- 0.69)
Abdominal obesity	0.53(0.45-0.61)	0.61(0.53- 0.69)
Elevated BP	0.54(0.46- 0.62)	0.64(0.56- 0.71)
Elevated triglyceride	0.62(0.54- 0.69)	0.66(0.58- 0.73)
Reduced HDL-c	0.58(0.50- 0.66)	0.73(0.65- 0.80)

Results are expressed as Area under Curve (confidence interval).

## Data Availability

Data is part of a composite project data and is therefore unavailable at the moment. Data will however be provided upon request.

## Abbreviations

MetS: Metabolic syndrome
HbA1c: Haemoglobin A1c
FPG: Fasting blood glucose
CVD: Cardiovascular disease
DM: Diabetes mellitus
SST: Serum separator tube
EDTA: Ethylene diamine tetraacetic acid
HDL-c: High density lipoprotein cholesterol
LDL-c: Low density lipoprotein cholesterol
VLDL-c: Very low-density lipoprotein cholesterol
HGI: Haemoglobin glycation index.
